# Comparison of *in vitro* acaricidal effects of commercial preparations of cypermethrin and fenvalerate against *Rhipicephalus (Boophilus) annulatus*

**DOI:** 10.1186/2193-1801-3-90

**Published:** 2014-02-14

**Authors:** Reghu Ravindran, Sunil Athalathil Ramankutty, Sanis Juliet, Adarsh Krishna Thumadath Palayullaparambil, Jyothimol Gopi, Ajith Kumar Karapparambu Gopalan, Suresh Narayanan Nair, Srikanta Ghosh

**Affiliations:** Department of Veterinary Parasitology, College of Veterinary and Animal Sciences, Pookode, Wayanad, Kerala 673 576 India; Department of Veterinary Pharmacology and Toxicology, College of Veterinary and Animal Sciences, Pookode, Wayanad, Kerala 673 576 India; Division of Parasitology, Indian Veterinary Research Institute, Izatnagar, Uttar Pradesh 243122 India

**Keywords:** Cypermethrin, Fenvalerate, *Rhipicephalus (Boophilus) annulatus*, Acaricidal activity

## Abstract

Commercially available preparations of cypermethrin (Clinar and Ectomin) and fenvalerate (Flytik and Ticomax, 20% E.C) were compared for their acaricidal activity against *Rhipicephalus (Boophilus) annulatus* using adult immersion test. Adult tick mortality was higher with Ectomin compared to Clinar. Complete eclosion blocking was observed at all the tested concentrations with Ectomin while it was observed only at the highest concentration tested for Clinar. Compared to Flytik, adult tick mortality was higher with Ticomax at the tested concentrations. Complete blocking of hatching of laid ova was observed with Flytik at the highest concentration tested. At the manufacture recommended dosage of 200 ppm Ectomin elicited 93.37 per cent inhibition of fecundity, while it was 91.7 per cent for Clinar. For fenvalerate, the recommended concentration was 1200 ppm at which Ticomax showed 86 per cent and Flytik produced 80.05 per cent inhibition of fecundity respectively.

## Introduction

Ticks are considered as one of the important and harmful blood sucking ecto-parasites of livestock around the world (Furman and Loomis 1984). Ticks produce several adverse effects and cause great economic loss to the livestock farmers (Snelson 1975). They transmit a wide variety of pathogenic agents and ranked second to mosquitoes in their vector potentiality (Oliver 1989). Globally, tick control is achieved mainly by the application of chemical acaricides. However, their indiscriminate use led to the development of acaricidal resistance in ticks (Martins et al. 1995). A number of non-organophosphate classes of pesticides have been developed which are effective against arthropod pests, environmentally safe and relatively less toxic to mammals and other non-target organisms when compared to OP compounds. Among these pesticides pyrethroids have excellent activity against several tick species, including *Boophilus* spp. (Davey and Ahrens 1984).

The common synthetic pyrethroids in use include deltamethrin, permethrin, cypermethrin, flumethrin and fenvalerate. Among this, the third generation pyrethroids, permethrin and fenvalerate, were the first of these available for ticks control on cattle (Davey and Ahrens 1984; Ware 2000). The present communication deals with the comparison of acaricidal effects of commercial formulation of two chemical acaricides *viz.*, Cypermethrin and Fenvalerate.

## Materials and methods

### Ticks

Fully engorged adult *Rhipicephalus* (*Boophilus*) *annulatus* female ticks were collected from infested cattle. The ticks were collected, washed with distilled water, dried using clean soft absorbent paper and used for the experiment.

### Chemical compound

Commercially available preparations of cypermethrin (Clinar and Ectomin, 10 per cent E.C.) and fenvalerate (Flytik and Ticomax, 20 per cent E.C.) were diluted in water to make different concentrations *viz.*, Clinar and Ectomin – 100 ppm, 200 ppm and 300 ppm, Flytik and Ticomax – 1100 ppm, 1200 ppm and 1300 ppm.

### Adult immersion test

Adult immersion test (AIT) was performed as per the protocol described by Drummond et al. (1973). Four replicates of six ticks each were used for testing of single dilution of each pesticide. Six ticks were immersed in the solution (10 ml) at room temperature for two minutes in a 25 ml beaker with gentle agitation. Water was used as control. Ticks were recovered from the solution, dried using absorbent paper and placed in separate plastic specimen tubes (25 mm × 50 mm). These tubes were incubated at 28 ± 1°C and 85 ± 5 per cent relative humidity in a biological oxygen demand (BOD) incubator.

Adult tick mortality was observed up to 15^th^ day post-treatment. After oviposition, the eggs laid by the female ticks were collected and weighed. Egg mass was observed under the same incubation conditions in a BOD incubator for the next 30 days. Ticks treated with different concentrations of the two compounds were compared with the control ticks.

The index of egg laying (IE) and percentage inhibition of fecundity (IF) were calculated (FAO 2004) as follows.

Index of egg laying/fecundity (IE/IF) = weight of eggs laid (g)/weight of females (g)

Percentage inhibition of fecundity (%IF) = [(IE control group – IE treated) × 100]/IE control group.

Hatching of eggs was observed visually.

### Statistical analysis

All the data were expressed as the mean ± SEM. Groups were compared using one-way analysis of variance for repeated measurements using SPSS software (IBM, USA). Fischer’s Least Square test and Duncan’s test were used for *post-hoc* analysis. A value of P < 0.05 was considered as statistically significant.

## Result and discussion

In the present study, two commercial preparations of cypermethrin (Clinar and Ectomin) and Fenvalerate (Ticomax and Flytik) were tested for their acaricidal activities.

Ectomin produced a higher level of mortality compared to Clinar at all tested concentrations (100, 200 and 300 ppm). A concentration dependent increase in adult tick mortality was observed for both preparations. However, both preparations could not produce 100 per cent mortality even at the highest concentration tested. The per cent inhibition of fecundity was higher with Clinar at 100 ppm concentration, while it was similar or slightly higher for Ectomin at 200 and 300 ppm concentrations. Complete eclosion blocking was observed at all the tested concentrations with Ectomin, while Clinar produced complete blocking only at the highest concentration tested (Table 1).

**Table 1:** **Effects of commercial formulations of cypermethrin and fenvalerate against** ***R. (B.) annulatus***

Active ingredient	Commercial pesticide	Concentration (ppm)	Mean ticks weight ± SEM	Mean% adult mortality within 15 days ± SEM	Mean eggs mass ± SEM	Index of fecundity ± SEM	Percentage Inhibition of fecundity (%)	Hatching % (Visual)
Cypermethrin	Ectomin	100 ppm	0.864 ± 0.044^cdef^	33.333 ± 9.624^bc^	0.114 ± 0.010^cd^	0.132 ± 0.009^de^	73.86	nil
		200 ppm	0.780 ± 0.0204^bc^	62.495 ± 4.165^def^	0.027 ± 0.027^ab^	0.034 ± 0.033^abc^	93.37	nil
		300 ppm	0.829 ± 0.0070^bcde^	66.66 ± 6.803^def^	0.011 ± 0.011^ab^	0.013 ± 0.013^ab^	97.39	nil
	Clinar	100 ppm	0.795 ± 0.0221^cde^	8.33 ± 4.809^ab^	0.058 ± 0.029^abc^	0.071 ± 0.033^abcd^	85.4	10
		200 ppm	0.797 ± 0.025^bcd^	20.827 ± 4.167^ab^	0.033 ± 0.018^ab^	0.041 ± 0.022^abc^	91.7	10
		300 ppm	0.862 ± 0.036^cdef^	33.33 ± 6.805^bc^	0.010 ± 0.014^ab^	0.012 ±0.001^ab^	97.6	nil
Fenvalerate	Flytik	1100 ppm	0.686 ± 0.057^a^	45.83 ± 12.50^cd^	0.079 ± 0.027 ^bcd^	0.108 ± 0.036^cde^	77.83	10
		1200 ppm	0.756 ± 0.037^ab^	49.997 ± 6.803^cde^	0.072 ± 0.018 ^abc^	0.097 ± 0.026^bcde^	80.05	10
		1300 ppm	0.782 ± 0.023^bc^	62.995 ± 4.165^def^	0.036 ± 0.019^ab^	0.045 ± 0.023^abc^	90.77	nil
	Ticomax	1100 ppm	0.847 ± 0.0402^bcde^	49.99 ±11.785^cde^	0.139 ± 0.062^d^	0.163 ± 0.069^e^	63.62	25
		1200 ppm	0.827 ± 0.009^bcde^	74.99 ± 15.958^ef^	0.052 ± 0.024^abc^	0.063 ± 0.028^abcd^	86	25
		1300 ppm	0.795 ± 0.014^bcd^	87.49 ± 7.980^f^	0.030 ±0.015^ab^	0.037 ± 0.019^abc^	91.66	10
Control		Water	0.885 ± 0.019^cdef^	0 ± 0^a^	0.442 ± 0.0065^e^	0.501 ± 0.010^f^	0	100

n = 4, Values are Mean ± SEM, means bearing different superscripts a, b, c or d (P < 0.05), indicate significant difference when compared with the control.

The effect on adult tick mortality was higher for Ticomax at all the tested concentrations (1100, 1200, 1300 ppm) when compared to Flytik. Both preparations showed a concentration dependent increase in adult tick mortality. However, 100 per cent mortality was not achieved even at highest concentration with any of these preparations. When compared to Ticomax, the per cent inhibition of fecundity was higher for Flytik at 1100 ppm while it was lower at 1200 and 1300 ppm concentrations. On visual examination, complete blocking of hatching of laid ova was observed for Flytik at all tested concentrations. Ticomax allowed hatching of the ova laid by the treated ticks to 10–25 per cent.

In insects, pyrethroids produce hyper-excitation and tremors, followed by paralysis. The nerve excitation occurs as a result of changes in nerve membrane permeability to sodium and potassium ions (Narahashi 1971). The insecticidal activity of pyrethroids mainly depends on the optical activity of the asymmetric carbon atom (Ruight 1985).

A study on the toxicity of cypermethrin and fenvalerate in different strains of *Drosophila melanogaster* revealed that cypermethrin was more toxic than fenvalerate at larval and adult stages (Alentorn et al. 1987). In the present study, higher inhibition of fecundity and absence of hatching of laid ova by treated *R. (B.) annulatus* were observed at concentration 300 ppm with both preparations of cypermethrin. This can be due to the fact that cypermethrin treatment produced an inhibition of development of ovary and reduction of both 20-hydroxyecdysone and vitellogenin (Vg) in the hemolymph of engorged ticks. In addition, the degree of salivary gland degeneration (which is triggered by 20E – [20-Hydroxyecdysone]) was slightly reduced in cypermethrin -treated engorged ticks (Friesen and Kaufman 2003).

Similarly cypermethrin treatment against *R. (B.) annulatus* resulted in the 100 per cent efficacy which sustained for up to 7 weeks post-treatment after which the efficacy was dropped and reached 98 per cent (Khalaf-Allah 1996). Also, the pour-on preparations of cypermethrin showed a higher *in vivo* efficacy compared to ivermectin against *Hyalomma anatolicum anatolicum* at 15 days post-treatment (Sajid et al. 2009).

In the present study, two commercial preparations of cypermethrin *viz.*, Clinar and Ectomin were tested. For both formulations the manufacturer recommends 2 ml solution per liter of water (200 ppm) for effective field application. For Flytik and Ticomax (20% E.C) the manufacturer recommends a dose rate of 6 ml/liter (1200 ppm) for field application.

The present paper, discusses the results of the *in vitro* experiments. Results of the present study revealed that to avoid development of any resistant generations of ticks in future, cypermethrin preparations should be used at or above 300 ppm while fenvalerate preparations should be used above 1300 ppm concentration. Hence, it is believed that when used *in vivo*, the dose rate should be either same or slightly more for getting the similar results.

## Conclusions

In the present study, commercial formulations of cypermethrin (Clinar and Ectomin) and fenvalerate (Flytik and Ticomax, 20% E.C) were compared *in vitro* for their acaricidal activity against *Rhipicephalus (Boophilus) annulatus* using adult immersion test. The commercial formulations showed variation in percentages of adult tick mortality, inhibition of fecundity and hatching of laid ova. Presently, manufactures recommend the use of cypermethrin at 200 ppm and fenvalerate at 1200 ppm. However, to avoid any development of resistant generation of ticks, cypermethrin formulations should be used at or above 300 ppm and fenvalerate formulations should be used at or above 1300 ppm.

## Abbreviations

AIT: Adult immersion test
BOD: Bio chemical oxygen demand incubator
°C: Degree celsius
EC: Emulsifiable concentrate
g: Gram
IE: Index of egg laying
IF: Index of fecundity
LC50: 50 per cent lethal concentration
ml: Millilitre
mm: Millimeter
OP: Organophosphate
ppm: Parts per million
SEM: Standard mean error
20E: 20-Hydroxyecdysone
Vg: Vitellogenin.
